# Arsenic impairs *Drosophila* neural stem cell mitotic progression and sleep behavior in a tauopathy model

**DOI:** 10.1093/g3journal/jkaf049

**Published:** 2025-04-07

**Authors:** Temitope H Adebambo, Fernanda Medina-Flores, Shirley Zhang, Dorothy A Lerit

**Affiliations:** Department of Cell Biology, Emory University School of Medicine, Emory University, Atlanta, GA 30322, USA; Department of Cell Biology, Emory University School of Medicine, Emory University, Atlanta, GA 30322, USA; Department of Cell Biology, Emory University School of Medicine, Emory University, Atlanta, GA 30322, USA; Department of Cell Biology, Emory University School of Medicine, Emory University, Atlanta, GA 30322, USA; Winship Cancer Institute, Emory University, Atlanta, GA 30322, USA

**Keywords:** arsenic, neurotoxicity, genome instability, neural stem cells, neurodevelopment, FlyBase

## Abstract

Despite established exposure limits, arsenic remains the most significant environmental risk factor detrimental to human health and is associated with carcinogenesis and neurotoxicity. Arsenic compromises neurodevelopment, and it is associated with peripheral neuropathy in adults. Exposure to heavy metals, such as arsenic, may also increase the risk of neurodegenerative disorders. Nevertheless, the molecular mechanisms underlying arsenic-induced neurotoxicity remain poorly understood. Elucidating how arsenic contributes to neurotoxicity may mitigate some of the risks associated with chronic sublethal exposure and inform future interventions. In this study, we examine the effects of arsenic exposure on *Drosophila* larval neurodevelopment and adult neurologic function. Consistent with prior work, we identify significant developmental delays and heightened mortality in response to arsenic. Within the developing larval brain, we identify a dose-dependent increase in brain volume. This aberrant brain growth is coupled with impaired mitotic progression of the neural stem cells (NSCs), progenitors of the neurons and glia of the central nervous system. Live imaging of cycling NSCs reveals significant delays in cell cycle progression upon arsenic treatment, leading to genomic instability. In adults, chronic arsenic exposure reduces neurologic function, such as locomotion. Finally, we show arsenic selectively impairs circadian rhythms in a humanized tauopathy model. These findings inform mechanisms of arsenic neurotoxicity and reveal sex-specific and genetic vulnerabilities to sublethal exposure.

## Introduction

The health implications of arsenic exposure are wide-ranging and multisystemic, affecting multiple organ systems and leading to neurologic, cardiovascular, and pulmonary dysfunction and cancer (Chen *et al*. 1988; Abdul *et al*. 2015). Arsenic is found in soil, food (e.g. rice and fish), and water worldwide. In the United States, arsenic levels within groundwater or soil can far exceed (5- to 7-fold) the maximum exposure limits set forth by the Environmental Protection Agency (10 parts per billion, ppb; ATSDR 2014). Human activities, including mining, pesticides, industrial applications, and smoking, increase risks of arsenic exposure (WHO 2022).

The World Health Organization (WHO) recognizes arsenic as a neurodevelopmental toxicant, particularly to children, affecting cognitive functions and developmental milestones (WHO 2022). Arsenic exposure severely impairs neurodevelopment, leading to lower IQ, cognition, and memory (Rodriguez-Barranco *et al*. 2013; WHO 2022). Consistent with these risks, arsenic is ranked number 1 on the Centers for Disease Control Agency for Toxic Substances and Disease Registry (ATSDR), prioritized on exposure risk and toxicity (ATSDR 2014, 2024). Its various oxidation states, especially As (III) and As (V), significantly influence the bioavailability and toxicity of arsenic (Watanabe and Hirano 2013).

The ability of arsenic and its metabolites to cross the blood–brain barrier, particularly in developing brains, promotes oxidative stress and cellular damage through mechanisms like mitochondrial dysfunction and apoptosis (Su *et al*. 2014; Niehoff *et al*. 2016). Neuronal responses are also altered by arsenic (Hong and Bain 2012; Chandravanshi *et al*. 2019). Despite the known risks of arsenic exposure, relatively little is known about the cellular mechanisms underlying arsenic neurotoxicity.

In addition to neurodevelopmental toxicity and neuropathy in adults, arsenic exposure may also contribute to the onset of neurodegenerative disorders, although this association is less understood (Pakzad *et al*. 2021; Rahman *et al*. 2021; Thakur *et al*. 2021). Alzheimer's disease and other dementias (ADRD) are neurodegenerative diseases causing progressive and irreparable neurologic deterioration and represent a major public health burden (Alzheimer's Disease Neuroimaging 2020). However, studies into how environmental exposures contribute to ADRD remain scarce. Phosphorylation of the microtubule-associated protein Tau (p-Tau) is a major component of neurofibrillary Tau-tangles, intracellular aggregates that are a primary biomarker and contributory factor to ADRD pathogenesis (Lee *et al*. 2004). In particular, the *Tau R406W* mutation is associated with early onset AD, frontotemporal dementia (FTD), and neurodegeneration (Wittmann *et al*. 2001). While there is some suggestion that exposure to heavy metals, such as As, may contribute to neurodegeneration (Pakzad *et al*. 2021), surprisingly little is known regarding the gene-by-environment interactions underlying ADRD.

Here, we examine the developmental and neurotoxic effects following exposure to sodium arsenite (NaAsO₂; hereafter, As) using *Drosophila* as a tractable model. We identify dosage-dependent effects of As-exposure on *Drosophila* neurodevelopment, viability, and behavior. We find these phenotypic responses vary by sex and genotype. Within the developing larval brain, As-exposure alters cell cycle progression of the neural stem cells (NSCs), which give rise to the neurons and glia of the adult brain. We find that sublethal As-exposure delays NSC mitotic progression, causing genomic instability. In adults, sublethal As-exposure reduces locomotion, consistent with diminished neurologic function. Finally, we assess the toxicogenetic responses of As-exposure by examining the effects of the *Tau^R406W^* tauopathy mutant associated with FTD on locomotor activity and sleep. Our findings indicate that the *Tau^R406W^* mutation impairs locomotion to a similar extent as arsenic, with a notable sex bias. Furthermore, *Tau* mutants are sensitized to arsenic toxicity, resulting in sex-specific sleep impairments.

## Materials and methods

### *Drosophila* stocks

The following strains and transgenic lines were used: *y^1^w^1118^* (Bloomington *Drosophila* Stock Center [BDSC] #1495) was used as the WT control, Oregon-R (BDSC stock #2057), *P(His2Av-mRFP1)II.2* (BDSC #23651) labels chromosomes with RFP (Pandey *et al*. 2005), *UAS*.*FUCCI* (BDSC #91704) is a reporter of cell cycle progression, *insc*-*GAL4 (*BDSC #8751) expresses GAL4 within NSCs under the *inscuteable (insc)* promoter, *elav*-*GAL4* (BDSC #8765) expresses GAL4 within neurons under the *embryonic lethal abnormal vision* (*elav*) promoter. *UAS*-*Tau^R406W^* (gift from Dr. Peng Jin, Emory University) expresses humanized Tau with the pathogenic R406W mutation (Wittmann *et al*. 2001). All lines were maintained on Bloomington formula cornmeal agar media (Lab-Express, Inc.; Ann Arbor, MI, USA) unless indicated and raised at 25°C in a light and temperature-controlled incubator.

### Preparation of As-containing medium

Defined concentrations of sodium arsenite (NaAsO₂; VWR International, cat# 97026-662) or ultrapure water for mock-treated controls were mixed into a custom medium containing 0.01% (w/v) tegosept (Genesee Scientific, #20-258), 5 g agar, and 12.5 g sucrose dissolved in 250 mL water. The medium was microwaved for approximately 2 min and then allowed to cool slightly before adding dilutions of a 10 mM liquid stock of NaAsO₂. For the adult acute toxicity assay, toxins were added to a fresh solution containing 4% (m/v) sucrose and 1.5% (m/v) dry yeast in ultrapure water.

### Arsenic exposure assays

#### Pupariation and eclosion

Zero to 4 h WT embryos were harvested and allowed to hatch into first instar larvae then transferred to vials with As-containing medium for a period of 14 days to model chronic exposure. Animals were daily monitored for pupariation or eclosion.

#### Adult acute toxicity assay and LD50 determination

As dose–response curves were performed on adult *y^1^w^1118^* and *Oregon-R Drosophila* strains aged 0–5 days as previously described (Holsopple *et al*. 2023). Flies were sorted by sex and subjected to partial starvation on grape juice agar plates for 16 h. After food deprivation, 20 males or females were seeded into separate vials containing liquid food (4% [m/v] sucrose and 1.5% [m/v] dry yeast powder) and different concentrations of As. One Kimwipe was inserted into each liquid food to prevent flies from drowning. To identify the approximate range of concentrations that can model As-exposure toxicity, flies were exposed to a range of As-concentrations (0, 0.01, 0.02, 0.1, 0.2, 1, and 2 mM). In refined experiments, 9 concentrations (0, 0.01, 0.15, 0.30, 0.45, 0.6, 0.75, 1, and 2 mM) were selected to more precisely define the As dose–response curve. Viability was scored at 24-h intervals for 48 h. To determine the LD_50_ values, the concentration required to kill 50% of the test animals, data from the 48-h mortality records were analyzed separately for male and female flies in both strains using the Environmental Protection Agency's publicly available Benchmark Dose Software version 3.3.2 available at https://bmdsonline.epa.gov/ (Agency 2023). Data were modeled as “dichotomous” and the Dichotomous Hill model was used for analyses using a benchmark response extra risk of 0.5 and 0.95 confidence limit.

#### Age-matched larval exposure

Age-matched third instar larvae were harvested, as described (Hailstock *et al*. 2023). Briefly, freshly eclosed adults (0–3 days old) were housed in acrylic collection cages to harvest 0–4 h embryos, which were incubated in a 25°C incubator for 24 h. A precision probe was used to transfer first instar larvae into a culturing vial supplemented with yeast paste containing 0.05% (w/v) bromophenol blue (Fisher Scientific, cat. no. BP115-25) supplemented with As (0, 5, or 10 μM). After 96 h, third instar larvae were selected based on complete gut clearance of blue yeast paste, corresponding to pupariation within 1–12 h in the control group.

### Isolation of *Drosophila* central nervous system

*Drosophila* larval brains were dissected as previously described (Lerit *et al*. 2014). Briefly, age-matched late third instar larvae, defined by complete food clearance from the gut, from both control and treatment groups were dissected in RT Schneider's medium (ThermoFisher Scientific, # 21720-024) on a glass slide under a dissecting microscope. Isolated intact brains were prepared for live imaging or transferred into a tube containing 0.5 mL Schneider's medium for immunofluorescence.

### Immunofluorescence

For immunofluorescence, samples were prepared as described (Lerit *et al*. 2014). Briefly, dissecting medium was removed, and samples were rinsed once with 0.5 mL of PBSTx (PBS supplemented with 0.3% Triton X-100), then fixed in 0.5 mL of 9% electron microscopy grade paraformaldehyde diluted in PBSTx at RT with nutation for 15 min. Fixative was removed, and the samples were washed 3 × 15 min with 0.5 mL PBSTx, blocked in PBT (PBS with 1% bovine serum albumin (BSA) and 0.1% Tween-20) for 1 h at RT with nutation, then incubated overnight at 4°C in 0.5 mL primary antibodies diluted in PBT supplemented with 4% normal goat serum (NGS), with nutation. The next day, samples were washed 3 × 15 min with 0.5 mL PBT, incubated for 1 h at RT in modified PBT (PBS, 2% BSA, 0.1% Tween-20, and 4% NGS), then for 2 h in secondary antibodies diluted into modified PBT. Samples were then washed 3 × 15 min with 0.5 mL PBST (PBS with 0.1% Tween-20), then manually oriented within a bubble of Aqua/Poly-mount mounting medium, which was left to polymerize overnight prior to imaging.

The following primary antibodies were used in this study: rabbit anti-phospho-Histone-3 (1:1000; Sigma-Millipore, 05-570), mouse anti-Repo (1:2000, DSHB, AB_528448), mouse anti-Elav (1:500, DSHB, AB_528217), rabbit anti-cleaved *Drosophila*-Dcp1 (Asp216) (1:500, Cell Signaling Technology, #9578) and rat anti-Miranda (1:500; Abcam, ab197788). Secondary antibodies were Alexa Fluor 488, 568, and 647 (1:500; Molecular Probes) and incubated with DAPI (10 ng/mL; ThermoFisher Scientific).

### EdU incorporation

We used the Click-iT EdU Cell Proliferation Kit (ThermoFisherScientific, Waltham, MA, USA, C10340) to assay EdU incorporation. Larval brains were isolated and incubated for 1 h in a tube containing 100 µM EdU diluted in Schneider's medium. Brains were then prepared for immunofluorescence, as above. EdU detection was performed after secondary antibody detection, according to the manufacturer’s instructions.

### Chromosomal preparations

Chromosomal spreads were prepared from WT third instar larval brains according to previously described methods (Pimpinelli *et al*. 2011). Briefly, brains from the treatment and control groups were dissected in 0.7% sodium chloride solution, transferred to a glass dissection dish containing 25 mM colchicine in 0.7% sodium chloride for 90 min, then incubated in 0.5% sodium citrate for 8 min. Samples were rinsed in a solution containing 11:11:2 methanol:acetic acid:water for 20 s, then incubated in 45% acetic acid for 2 min prior to being squashed on glass microscope slides. The slides were transferred to dry ice, washed in pre-chilled −20°C ethanol, then allowed to air-dry. Spreads were immediately rehydrated in 2X SSC (saline-sodium citrate buffer) before staining with DAPI for 5 min, followed by gentle rinsing with 2X SSC, and mounting in Aqua/Poly-mount. Chromosomes were scored from at least 35 NSCs per condition.

### Microscopy

All images were acquired on a Nikon Ti-E inverted microscope using a Yokogawa CSU-X1 spinning disk head (Yokogawa Corp. of America), Orca Flash 4.0 v2 CMOS camera (Hamamatsu Corp.), and Nikon LU-N4 solid-state lasers (15 mW; 405, 488, 561, and 647 nm) using the following Nikon objectives: 100 × 1.49-NA Apo Total Internal Reflection Fluorescence oil immersion, 40 × 1.3-NA Plan Fluor oil immersion, and 20 × 0.75-NA Plan Apo.

### Live imaging

*Drosophila* larval brains were dissected as described (Lerit *et al*. 2014), except the Schneider's medium was supplemented with 0.1% glucose. Brains were prepared for imaging following the clotting method, as described (Pampalona *et al*. 2015). Briefly, following removal of imaginal discs, brains were placed in a 2 µL drop of 10 mg/mL fibrinogen dissolved in medium in the center of a 35 mm glass bottom dish (MatTek Corporation, Ashland, MA, USA, P35G-1.5-14-C). 1.5 µL of thrombin was added, allowing the mixture to clot in the dark for 2 min. The procedure involving the addition of fibrinogen and thrombin was repeated once more, and the resulting clot was then covered with 600 µL of glucose-supplemented medium. Samples were imaged in the dark at RT.

Images were acquired at 25°C with a 40X 1.3 NA oil objective using ∼1 μm Z-stacks across a total depth of ∼20 μm at 1-min intervals over a duration of 181 min. Microscope settings were controlled through Nikon Elements AR software on a 64-bit HP Z440 workstation (Hewlett-Packard).

### Image analysis

For the following analyses, the experimenter was blinded to the genotype/condition by anonymizing control and experimental file names using a custom macro. For *brain volume*, the total 3D volume of a single optic lobe (OL) or a specified region was measured in Imaris 10.1 software (Oxford Instruments) using the 3D surface tool. The CB and OL sub-regions were manually defined and the statistics function was used to calculate surface volume. Here, the neuroepithelium ridge served as a boundary for measuring the CB vs OL regions. The CB resides in the medial half of the larval brain and is noted by the large NSC nuclei and associated clusters of smaller progeny cells. The OL comprises the lateral half and contains cells from the neuroepithelium, lamina, and the inner and outer proliferation centers (Weng *et al*. 2012). *pH3+ cells* were quantified using an existing pipeline (3D Noise Nuclei segmentation) from Cell Profiler 4.24 (McQuin *et al*. 2018). *EdU incorporation* was quantified in Fiji by manually scoring EdU+/Mira+ central brain NSCs across optical sections. Repo+, Miranda+, and Elav+ cells were analyzed for cell number and cell volume using the Labkit machine learning plugin and 3D object counter in Fiji (Arzt *et al*. 2022). Percent overlap between Dcp1 + cells and each of Repo+, Miranda + and Elav + cells were quantified by manual scoring. *Cell cycle duration* was measured from live imaging successive anaphase-onset events, defined by the oriented separation of the chromosomes to the poles.

Images were cropped, channels separated, and LUTs adjusted using Fiji and Adobe Photoshop software. Figures were assembled in Adobe Illustrator.

### Behavioral assays

For the *negative geotaxis assay* (*NGA*),10 males or virgin females aged 0–3 days were seeded into vials with As-containing medium corresponding to fractions (1/50th and 1/10th concentration) of the sex-specific As-concentrations determined by early estimations of the *yw* LD50 values and a control group and reared at 25°C for 7 days to model chronic arsenic exposure. Subsequently, the flies were transferred to vials containing an agarose pad (2% agarose and 5% sucrose diluted in 2 mL of ultrapure water). Up to 4 vials were fitted into a custom 3D-printed holder, tapped down once, and recorded with a Panasonic HC-V800 digital video recorder at 60 frames per second to monitor climbing activity, as described (Behnke *et al*. 2021). Videos were viewed in VLC Media Player (https://www.videolan.org/vlc/), and the number of flies that successfully crossed a 10 cm mark within 10 s after tapping were manually counted. The NGA was repeated for 5 additional biological replicates containing 10 flies each, and measurements are displayed as pooled across replicates.

For *sleep behavior* analysis, male and virgin flies aged 0–7 days and subjected to As-treatment, following the same protocol as the NGA, were loaded into 65 mm × 5 mm glass locomotor tubes containing *Drosophila* culturing medium. The flies had the opportunity to acclimatize during the first day. Data from days 2, 3, and 4 were collected to perform sleep activity analysis using a single beam *Drosophila* Activity Monitoring System with DAM2 monitors (TriKinetics, Waltham, MA). Sleep was defined as bouts of uninterrupted inactivity lasting for ≥5 min (Hendricks *et al*. 2000; Shaw *et al*. 2000). Sleep parameters (total sleep, day sleep, night sleep, bout number, activity index, and sleep tracers) were analyzed for each 24-h period and averaged across 3 days. Sleep analysis was conducted in 1-min bins using the MATLAB-based program PHASE (Persons *et al*. 2022).

### Statistical analysis

Statistical analysis and data plotting were conducted using GraphPad Prism (ver. 9). Data were subjected to a D′Agnostino and Pearson normality test. Outliers were identified by the GraphPad ROUT outlier analysis using the default settings. Data were then subjected to an unpaired *t*-test, 1-way ANOVA, or the appropriate non-parametric test. Error bars depicted in all figures signify mean ± standard deviation (SD). The displayed data are representative of at least 2 independent experiments, as indicated in the figure legends.

## Results

### Arsenic toxicity in *Drosophila melanogaster*

**Fig. 1.**
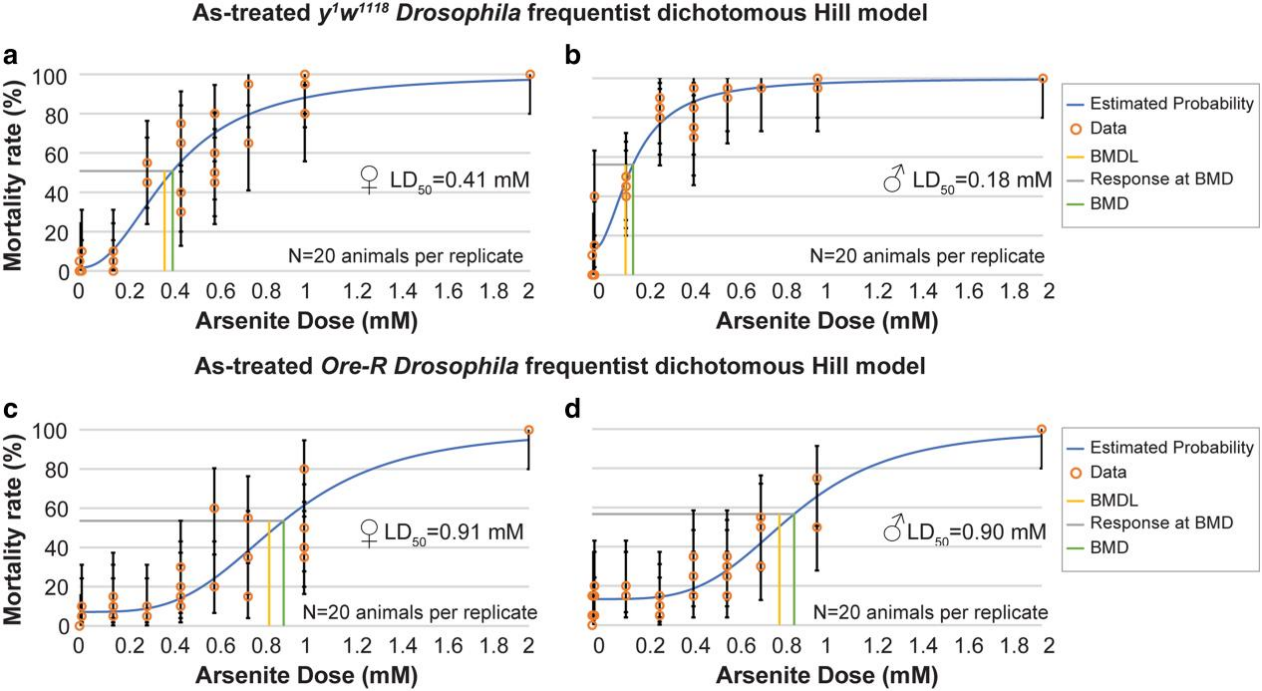
Dose–response curves for *Drosophila* adults exposed to As. Graphs show the estimated mortality rate for a–b) *yw* and c–d) *Ore-R* adults. A broad range of As-exposure was tested in the finding experiments to approximate when 50% of adults die (LD50). A narrower range of doses was tested in the refined experiments shown in a–d). For all conditions, *N* = 4 vials with 20 adults per vial. Data are displayed as mean ± S.D. BMD, benchmark data; BMDL, benchmark data lower confidence limit as calculated using the Benchmark Dose Software (BMDS) from the Environmental Protection Agency, v. 3.3.2 (see *Methods*).

We first examined how acute As-toxicity is impacted by genetic background using 2 common control laboratory strains: *y^1^w^1118^* (hereafter, *yw*) and *Oregon-R* (hereafter, *Ore-R*). For these experiments, we followed the method of Holsopple *et al*., including an initial range-finding experiment to approximate the lethal dose 50 (LD50), the dose of As at which 50% of adult *Drosophila* die (Holsopple *et al*. 2023). Several other As doses were subsequently tested in a refined experiment to calculate the LD50 using benchmark data from the Environmental Protection Agency (Agency 2023) (Fig. 1). The data indicate the LD50 for *yw* females (0.41 mM) is about 2-fold higher than *yw* males (0.18 mM; Fig. 1, a and b). By comparison, the LD50 for *Ore-R* females (0.91 mM) and males (0.90 mM) was higher than *yw* (Fig. 1, c and d). These data indicate that *yw* adults are more sensitive to acute As-exposure than *Ore-R*, consistent with prior work showing differential toxicity of As across various *Drosophila* isolates (Ortiz *et al*. 2009). These data also highlight a sex-specific vulnerability to As-exposure, with male mortality occurring at lower concentrations than females, as recently shown (Holsopple *et al*. 2023). Because *yw* is more sensitive to As-exposure, we used this genetic background for the remainder of our study, unless otherwise noted.

**Fig. 2.**
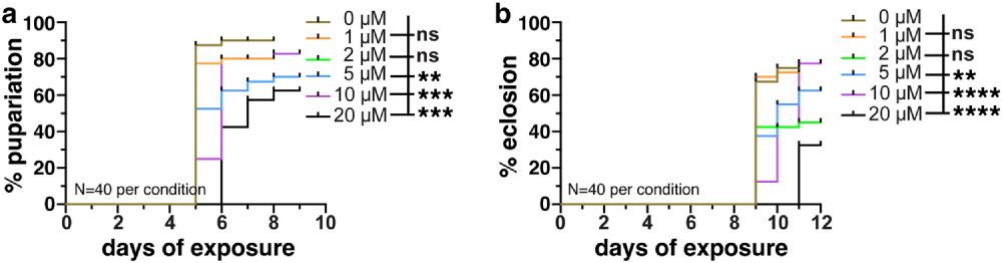
As-exposure impairs development. a) Dose response of chronic As-exposure on pupariation shows concentrations ≥ 5 μM elicit a delay in the developmental transition from larval to pupal stages, as compared with controls. b) Similar delays in adult eclosion were noted. *N* = 40 adults per condition for each trial. Statistical significance by 1-way ANOVA. ****P ≤ 0.0001, ***P ≤ 0.001, **P ≤ 0.01; ns, not significant.

We next examined how chronic exposure to sublethal concentrations of As impacted development. Chronic As-exposure resulted in a concentration-dependent inhibition of pupariation and eclosion (Fig. 2, a and b). Exposure to 5 μM As or greater delayed, and eventually arrested, developmental progression. By 10 days after exposure, nearly all larvae in the control and low-exposure groups had pupariated (defined by larval cuticle formation and thickening). In contrast, larvae exposed to ≥ 5 µM As exhibited significantly reduced pupariation rates (Fig. 2a).

Eclosion of the mature adult from the pupal case was similarly delayed. While eclosion rates did not differ from the control at lower concentrations, 5 μM As or greater resulted in a notable delay. This inhibitory effect on eclosion was further amplified at higher concentrations and sustained throughout the treatment period (Fig. 2b). Taken together, our findings illustrate dose-dependent impairments to developmental progression and viability in adult *Drosophila* following As-exposure, consistent with prior work (Anushree *et al*. 2023).

### Arsenic alters *Drosophila* neurodevelopment

**Fig. 3.**
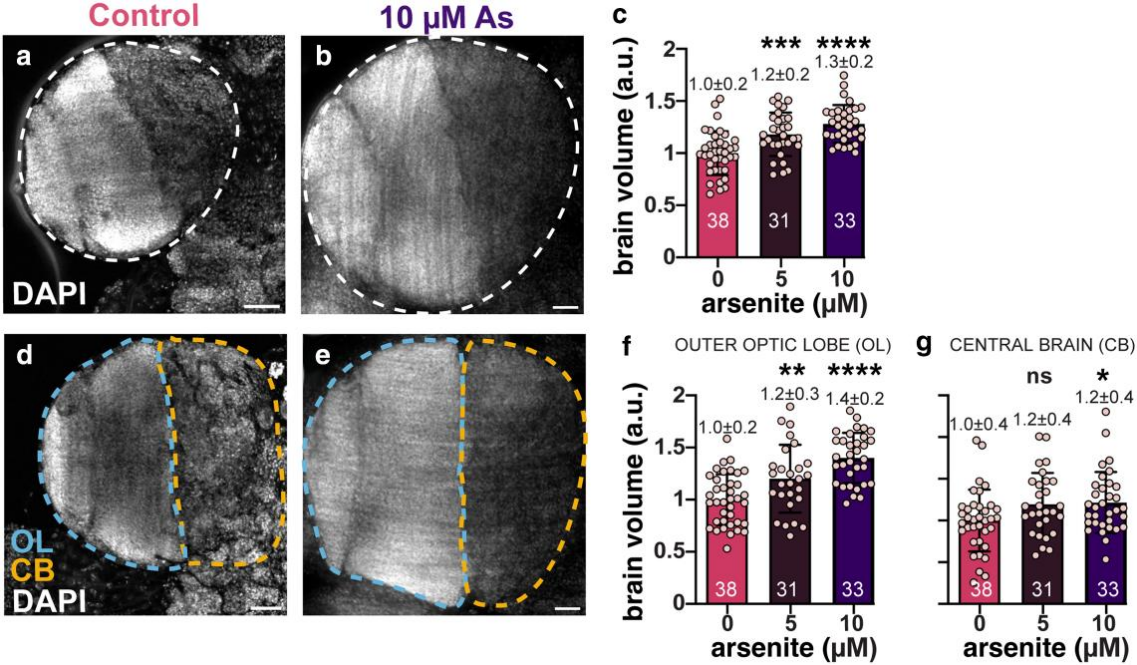
Chronic As-exposure causes larval brain hypergrowth. Representative maximum intensity projections of a) control and b) As-exposed third larval instar brains stained with DAPI (gray). c) Volumetric analysis of larval brains, where each dot represents a single measurement from 1 OL from *N* = 38 untreated, 31 0.5 µM As, and 33 10 µM As-treated samples. Volume scales as a dose-dependent effect. d and e) The OL was divided into 2 regions, the outer OL (blue line) is the lateral region comprising the neuroepithelium, medulla, outer proliferation center, etc. vs the medial CB (orange line). f) OL and g) CB volumes trend upwards following As-exposure. The experiment was repeated in triplicate. Mean ± SD indicated. Significance determined by 1-way ANOVA; ****P ≤ 0.0001, ***P ≤ 0.001, **P ≤ 0.01, *P ≤ 0.05; ns, not significant. Scale bars = 30 μm.

We next sought to investigate the mechanistic basis of As-induced neurotoxicity. Larval development marks an important phase of *Drosophila* neurogenesis, giving rise to most of the neurons of the adult brain (Homem and Knoblich 2012). Sublethal exposure to As led to significantly enlarged larval brain volumes in a dose-dependent manner (20–30% increase; Fig. 3, a–c).

The *Drosophila* larval brain is organized into morphologically distinct regions defined by cellular lineages. For example, the central brain (CB) is abundant in the highly proliferative type I and II neuroblasts, or NSCs (Homem and Knoblich 2012). To determine if As differentially affected specific larval brain regions, we compared the CB and the remaining OL volumes (see *Methods*) from treated vs control groups. While both the CB and OL regions were enlarged following As-exposure, the OL was more sensitive. In particular, the OL volume increased by 40% with 10 µM-As (****P ≤ 0.0001 by ANOVA; Fig. 3, d–g). These data reveal a dose-dependent volumetric increase in the OL and CB following As-exposure and underscore the regional specificity in the brain's response to toxic insults.

### Arsenic impairs cell cycle progression

**Fig. 4.**
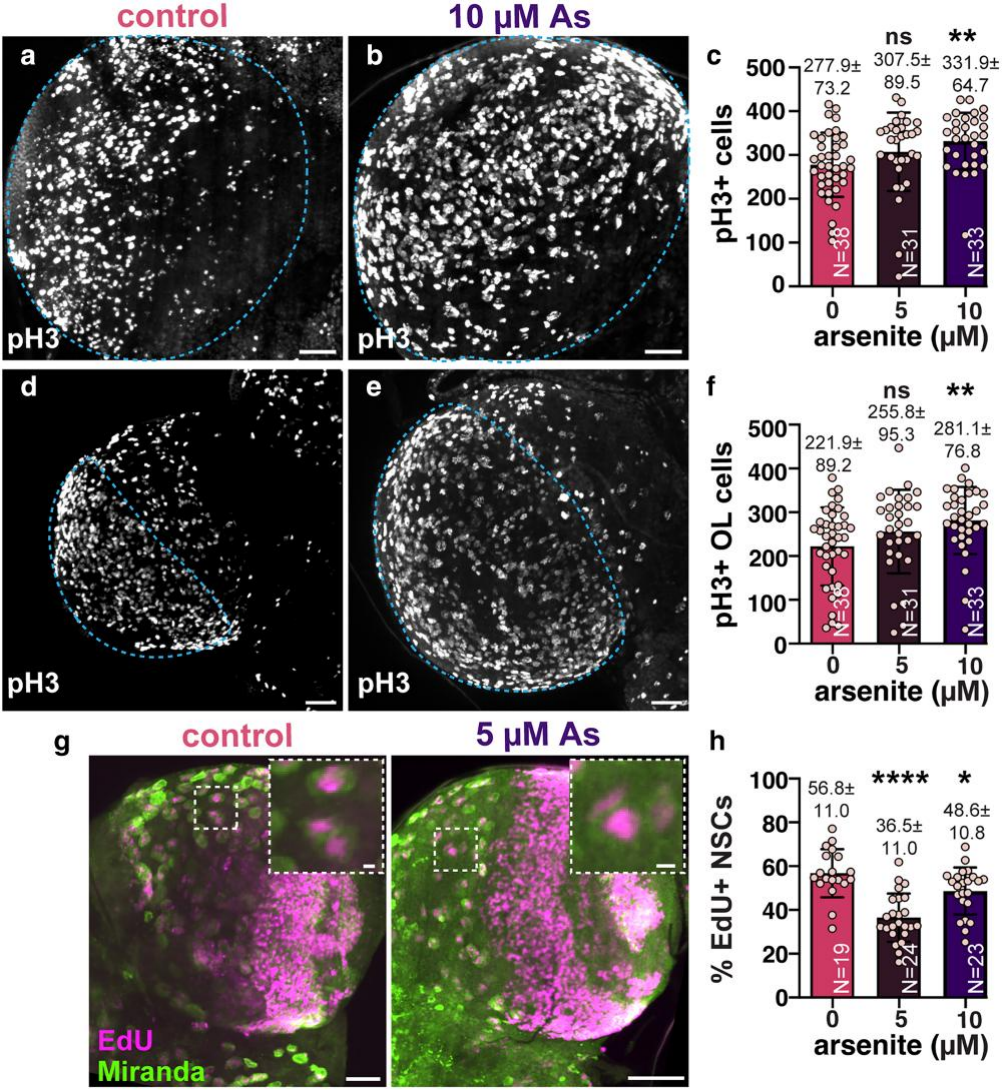
Chronic As-exposure alters cell cycle dynamics. Maximum intensity projections of a) control and b) As-exposed third larval instar brains marked with pH3 to label mitotic cells. c) Quantification of pH3+ cells; each dot represents a single measurement from 1 brain from *N* = 38 untreated, 31 0.5 µM As, and 33 10 µM As-treated samples from 2 replicates. Mitotic activity was assessed within the OL of d) control vs e) As-exposed brains. f) Quantification of pH3+ cells in the OL. g) Control or 5 µM As-exposed brains stained with Mira (green) to label NSC and EdU (magenta) to monitor DNA synthesis. Boxed regions indicate insets. h) Quantitation of EdU + central brain NSCs reveals reduced DNA synthesis in treated vs control groups. *N* = 19 untreated, 24 0.5 µM As, and 23 10 µM As-treated samples from 2 replicates. Mean ± SD indicated. Significance by 1-way ANOVA; ****P ≤ 0.0001, **P ≤ 0.01, *P ≤ 0.05; ns, not significant. Scale bars = 30 μm; insets, 10 μm.

To determine if the observed increase in brain volume following As-exposure was due to elevated rates of cell division, we first quantified the number of cells positive for the pro-mitotic marker phospho-Histone H3 (pH3). As-treatment increased the number of pH3 + cells within larval brains by 20% following 10 µM-As-exposure (**P ≤ 0.01 by ANOVA; Fig. 4, a–c), demonstrating As-exposure deregulates cell proliferation. A similar elevation in pH3 + cells was also observed within the OL region, where volume was most significantly increased (Figs. 3f and 4, d–f). These enhanced rates of cell division may contribute to the enlarged brains resulting from As-exposure.

To test if the elevated volumes observed in the CB were due to increased NSC divisions, we monitored rates of EdU incorporation in treated vs control samples. Unexpectedly, we observed a reduction in EdU + NSCs following chronic As-exposure (Fig. 4, g and h). Together, these results show As alters the cell cycle progression and are consistent with earlier work identifying a G1/S and G2/M block in As-exposed cancer cell models (Sheldon 2017).

**Fig. 5.**
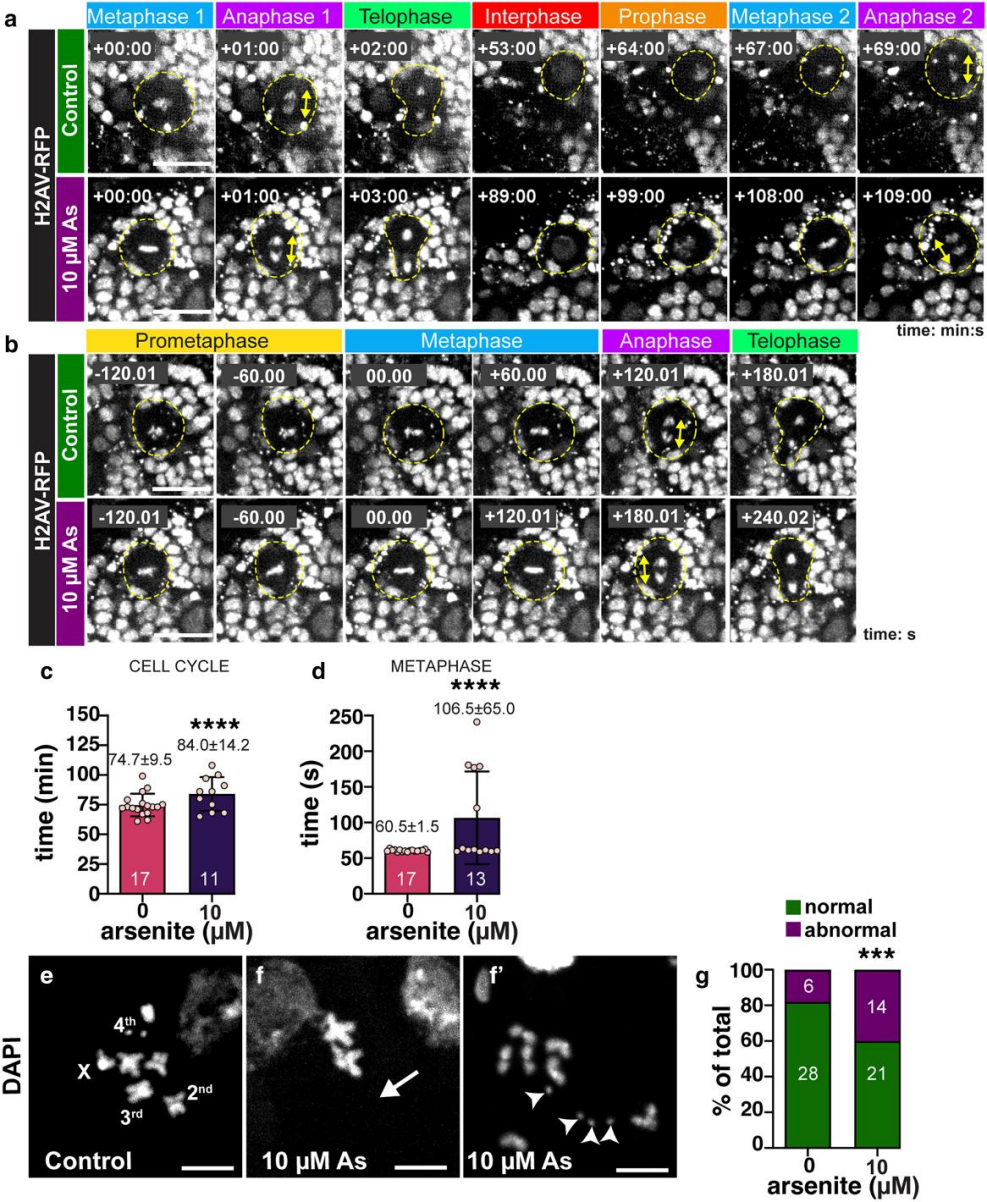
Errant cell cycle progression in As-exposed NSCs. Stills from live imaging of a) control or b) As-exposed larval brains expressing *H2AV-RFP*. Cycling NSCs are highlighted (dashed circle) and anaphase-onset is marked by the double-headed arrows. Time 0:00 is relative to the first metaphase-onset. a) Two successive NSC divisions are shown with time displayed as min:s. Video 1 shows a control cycling NSC. Video 2 shows a cycling As-exposed NSC. b) A single NSC division is shown with time displayed in s. Video 3 shows a control NSC. Video 4 shows an As-exposed NSC. c) Quantification of total cell cycle duration (min) from *N* = 17 control and 11 As-treated (10 µM) samples. d) Time spent in metaphase (s) from *N* = 17 control and 13 As-treated (10 µM) samples. Chromosome spreads show the karyotype of e) control vs f and f′) 10 µM As-treated NSCs. The 4 chromosomes are labeled; arrow marks whole chromosome loss, while arrowheads denote chromosomal gains. g) Quantification of aneuploidy from *N* = 34 control and 35 As-exposed NSCs. For each experiment, *N* = 5 brains were imaged across 5 replicates. Mean ± SD indicated. Significance determined by c and d) unpaired *t*-test and g) Fisher's exact test; ****P ≤ 0.0001 and ***P ≤ 0.001. Scale bars = a and b) 10 μm; e–f′) 5 µm.

Given the altered frequency of EdU + vs pH3 + cells, we reasoned As may cause cells to stall in mitosis. To test this hypothesis, we live imaged mitotic progression in cycling NSCs from age-matched third instar larvae expressing *H2Av-RFP*, which labels chromosomes. The total cell cycle length of NSCs became significantly lengthened following As-exposure (Fig. 5, a and c). Over the course of these experiments, we noted that a subset (∼40%) of As-exposed NSCs exhibited persistence of the metaphase plate relative to controls (Fig. 5, b and d). These As-exposed NSCs spend about 75% more time in metaphase than control cells (Fig. 5d), contributing to their extended cycling time.

Defects in cell cycle progression are often associated with genomic instability and aneuploidy (Gatti and Baker 1989; Lange *et al*. 2000). We therefore examined chromosomal preparations from larval brains to assess genome integrity in control vs As-exposed samples. While controls showed well-arranged euploid mitotic figures (Fig. 5e), As-exposure resulted in elevated rates of aneuploidy, including whole chromosomal loss (*arrow*, Fig. 5f), or gain (*arrowheads*, Fig. 5f′). Approximately 40% of the mitotic figures examined in the treatment group showed aberrant mitotic figures, significantly more than controls (Fig. 5g; *P* = 0.00107 by Fisher's exact test). Taken together, these results suggest that As delays cell cycle progression, likely due to a failure in the spindle assembly checkpoint, resulting in aneuploidy.

### Response of neurogenic lineages to arsenic

**Fig. 6.**
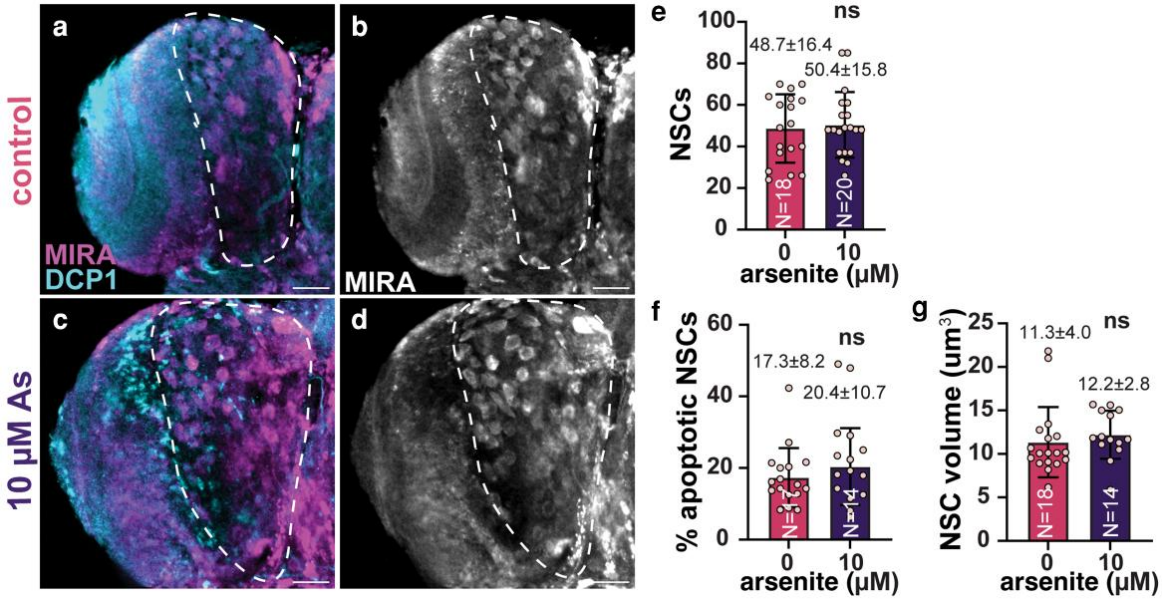
Maintenance of NSCs upon sublethal As-exposure. Representative maximum intensity projections of a and b) control and c and d) As-exposed (10 µM) NSCs stained with Mira (magenta) and DCP-1 (blue) antibodies. e) Quantification of NSCs from *N* = 18 control and 20 As-treated (10 µM) third instar larval brains. f) The percentage of apoptotic (DCP+) NSCs from *N* = 17 control and 14 As-treated brains. g) Volumetric analysis of *N* = 17 control and 14 As-treated NSCs. For e and f), each dot represents a measurement from a single brain. For g), each dot is an averaged measurement of NSC volume from a single brain. Significance by Student's *t*-test; ns, not significant. Scale bars = 30 μm.

Mitotic errors and elevated rates of aneuploidy within NSCs may lead to cell death (Poulton *et al*. 2017). However, we found no difference in the number of NSCs within untreated controls relative to As-exposed samples (10 µM), nor did Dcp-1 (death caspase-1) staining suggest elevated rates of apoptosis (Fig. 6, a–g). These data argue that the aneuploidy associated with As-exposed NSCs is unlikely to cause cell death. Other models of aneuploidy similarly failed to induce cell death within NSCs, suggesting robust mechanisms protect NSCs from apoptosis (Gogendeau *et al*. 2015; Mirkovic *et al*. 2019).

**Fig. 7.**
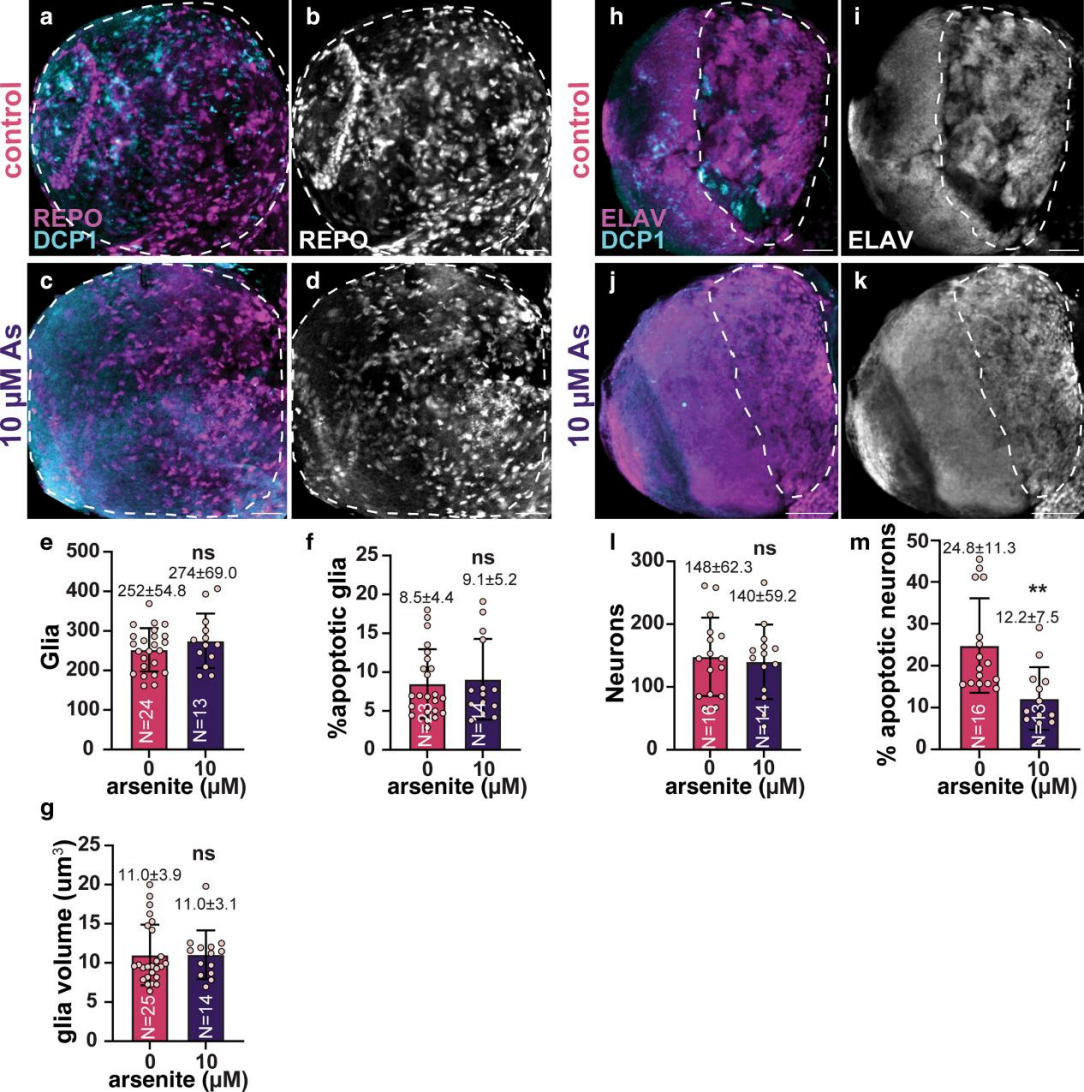
Effects of sublethal As-exposure on glia and neurons in larval brains. Representative maximum intensity projections of control and As-exposed (10 µM) third larval instar brains stained for DCP-1 (blue) and the indicated antibodies. Anti-Repo antibodies were used to label glia in a and b) control and c and d) As-exposed (10 µM) brains. e) Quantification of glia from *N* = 23 control and 14 As-exposed samples. f) The percentage of apoptotic (DCP+) glia from *N* = 23 control and 14 As-treated brains. g) Volumetric analysis of *N* = 25 control and 14 As-treated glia. For e and f), each dot represents a measurement from a single brain. For g), each dot is an averaged measurement of glia volume from a single brain. Anti-Elav antibodies were used to label neurons in h and i) control and j and k) As-exposed (10 µM) brains. l) Quantification of neurons from *N* = 16 control and 14 As-exposed samples. m) The percentage of apoptotic (DCP+) neurons from *N* = 16 control and 13 As-treated brains. Each data point in l and m) represents a measurement from a single brain. Significance by Student's *t*-test; **P ≤ 0.01; ns, not significant. Scale bars = 30 μm.

NSCs undergo asymmetric cell division to self-renew and generate the neurons and glia required for neural development (Homem and Knoblich 2012). We therefore examined the number of glia or neurons within control vs As-exposed brains. We noted no significant difference in glia number, rates of apoptosis, or volume (Fig. 7, a–g). We similarly failed to detect a loss of neurons or an increase in neuronal death (Fig. 7, h–m). We conclude that higher concentrations of As are required to induce neuronal death (Feng *et al*. 2024). Taken together, our analysis suggests that the increase in larval brain size associated with sublethal As-exposure may be independent of an increase in NSCs, glia, or neurons.

### Arsenic impairs locomotion to a similar extent as a humanized tauopathy model

**Fig. 8.**
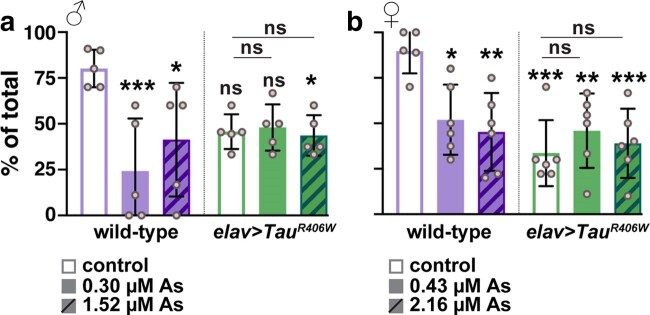
As-exposure impairs locomotor activity. Quantification of the NGA to measure climbing behavior. The *y*-axis displays the percentage of animals that cross a 10 cm mark within 10 s, and each dot represents the average response of 10 individuals. WT or *elav > Tau^R406W^* males or virgin females were exposed to the indicated As-concentrations, adjusted for sex. a) Climbing activity in WT males was significantly reduced upon As-exposure. Although *Tau^R406W^* males showed reduced climbing relative to WT, this only reached significance with the highest dose of As tested. b) Climbing activity in WT virgin females showed a dose-dependent decline with As-exposure. Conversely, *Tau^R406W^* females exhibited similar impairments to climbing activity in the presence or absence of As-exposure. Mean ± SD indicated. Significance is determined by ANOVA; ***P ≤ 0.001; **P ≤ 0.01; *P ≤ 0.05; and ns, not significant.

Given our findings that low concentrations of As impair neurodevelopment, we next assayed their neurologic consequences. To assay locomotor behavior, we examined climbing activity through a negative geotaxis assay (NGA). For these experiments, we examined the dose-dependent effects of a low As-exposure, adjusted by sex, relative to another 5-fold greater dose (Fig. 8). About 80–90% of untreated WT control flies completed the climbing task within 10 s. In contrast, As-exposure significantly impaired locomotion in males and virgin females by about 50%, consistent with decreased neurologic function (Fig. 8, a and b). These data are consistent with prior work showing reduced climbing activity in response to significantly higher doses of As (Anushree *et al*. 2023).

We next sought to compare the As-induced deficit to climbing activity in the established tauopathy model, *Tau^R406W^* (*elav > UAS-Tau^R406W^*) (Wittmann *et al*. 2001). While some reports suggest that As-exposure increases susceptibility to neurodegenerative disorders, the toxicogenetic interaction of As with genetic risk factors remains poorly studied (Pakzad *et al*. 2021). Untreated *Tau^R406W^* mutants displayed a ∼40–70% reduction in climbing activity relative to WT, consistent with prior reports (Wittmann *et al*. 2001; Beharry *et al*. 2013; Nikookar *et al*. 2021). Female *Tau* mutants performed the NGA worse than their male siblings, marking a sex-specific bias in the extent of Tau-mediated climbing impairment (Fig. 8, a and b). We then examined whether *Tau* mutants were susceptible to As-exposure. Our findings revealed no significant difference in climbing activity between untreated vs treated *Tau* mutants (Fig. 8, a and b). Relative to untreated WT controls, As-exposure and the *Tau^R406W^* mutation decrease locomotor activity to similar degrees. While our findings do not support a toxicogenetic interaction in this context, higher doses of As may yield different results.

### *Tau* mutants are sensitized to As-mediated sleep disturbances

**Fig. 9.**
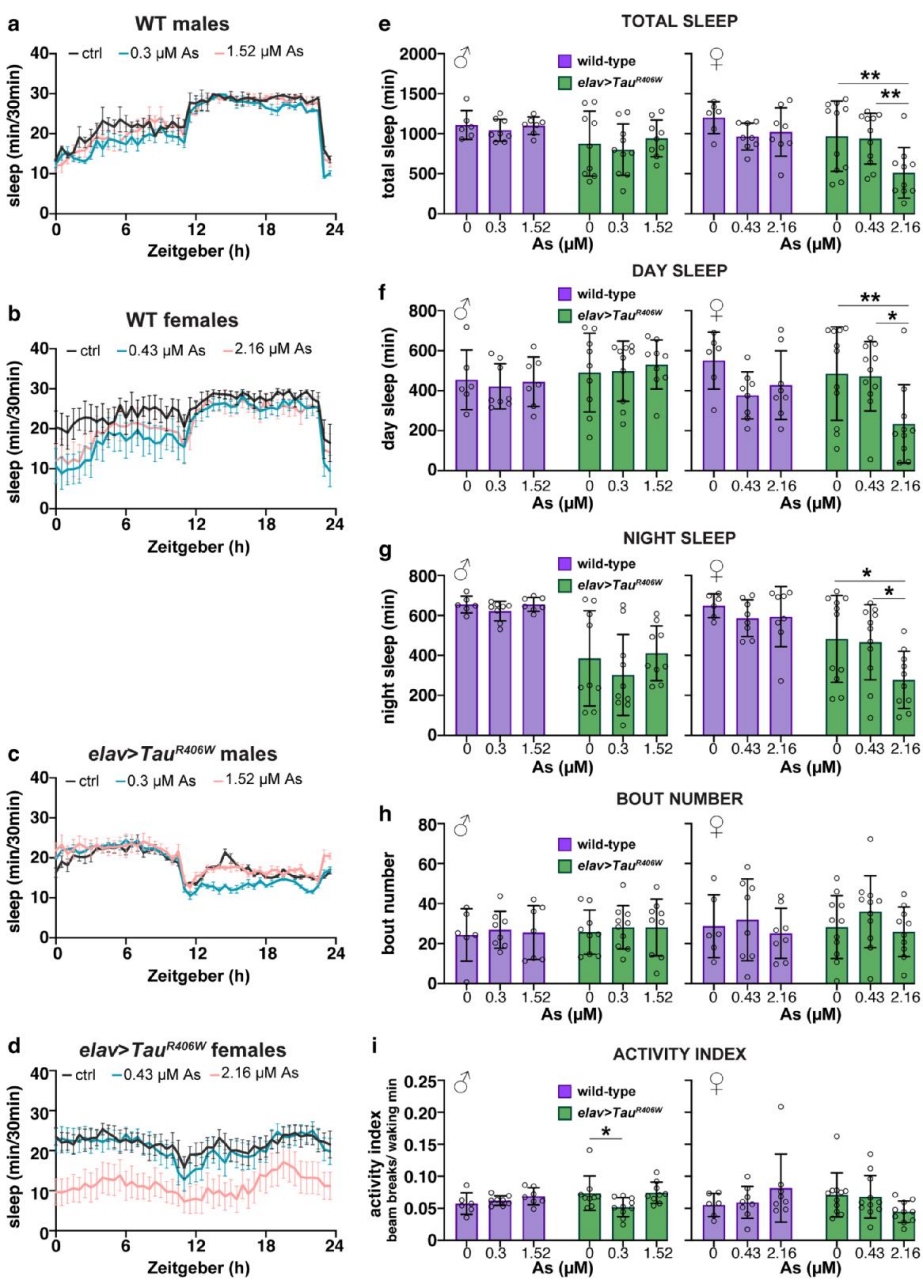
Diminished sleep behaviors in As-exposed *Tau* mutants. Traces of sleep/wake activity from 0 to 7 days old a) WT males, b) WT virgin females, c) *Tau^R406W^* males, or d) *Tau^R406W^* virgin females. Adults were either mock-treated (black) or exposed to a lower (blue) or 5-fold higher sex-specific dose. Flies were reared in a 12-h LD cycle in a light-controlled DAM (see *Methods*). e–i) Quantification of sleep parameters from males and virgin females of the indicated genotypes and treatment groups. Each dot represents the response of a single individual. Only As-treated *Tau^R406W^* mutants showed a significant difference in sleep behaviors: e) total sleep, f) day sleep, and g) night sleep were reduced in *Tau^R406W^* females exposed to 2.16 µM-As. i) *Tau^R406W^* males exposed to 0.3 µM-As had a lower activity index, while the 1.52 µM group did not. No significant changes in circadian rhythms were detected in the As-treated control (WT) samples. *N* = 6–11 individuals per genotype/treatment group from 2 independent experiments. Data plotted as mean ± SD. Significance determined by 2-way ANOVA followed by Tukey's multiple comparisons test with **P ≤ 0.01 and *P ≤ 0.05. All other values were not significantly different from the respective untreated control.

A defined subset of neurons within the brain regulates the cyclic pattern of sleep or activity known as circadian rhythms. During a typical 24-h light/dark cycle (12-h light: 12-h dark), WT *Drosophila* normally display patterns of daytime activity and nighttime inactivity, or sleep (Hendricks *et al*. 2000; Shaw *et al*. 2000). Using a *Drosophila* activity monitor (DAM) to measure the number of times an individual crosses an infrared beam, we examined various sleep parameters in WT animals with or without As-exposure (Fig. 9, a and b). This assay revealed no significant differences in total sleep, day- or nighttime sleep, sleep bout number, or activity index in untreated control males or virgin females vs those exposed to sex-adjusted As-concentrations (Fig. 9, e–i).

In contrast, we detected a sex-specific and As-dependent sleep impairment in *Tau^R406W^* mutants (Fig. 9, c and d). Total sleep, day sleep, and night sleep were all reduced in *Tau^R406W^* females exposed to As relative to their unexposed counterparts (Fig. 9, e–g). *Tau^R406W^* males also exhibited a lower activity index with As, although this response was not consistent in the higher treatment group and no other sleep parameters were affected (Fig. 9i). The increased restlessness observed in *Tau* mutant females indicates that this genetic background is sensitized to As-mediated neurotoxicity.

## Discussion

Although extensive research has established arsenic exposure as a potent human health concern implicated in carcinogenesis and neurotoxicity, the underlying cellular mechanisms remain inadequately understood. While previous studies in *Drosophila* have primarily established the dose-dependent lethality of arsenic (Meyer *et al*. 2014; Anushree *et al*. 2023), our findings illuminate significant sex, genotype, and dose-dependent impairments to neurodevelopment, viability, and adult behavior.

Here, we examined cellular responses within the developing brain following As-exposure. Unexpectedly, we noted increased larval brain volumes. Moreover, enlarged brain size was associated with disrupted cell cycle progression marked by more cells in mitosis and fewer cells in S-phase. Consistent with these data, live imaging of cycling NSCs revealed As-treated NSCs take longer to divide. We speculate the prolonged metaphase evident in As-exposed NSCs is likely due to a failure to satisfy the SAC, resulting in elevated rates of genomic instability. Our finding that the increase in larval brain volume associated with As-exposure was not associated with an increase in NSCs, glia, or neurons may reflect a limitation of spinning disk microscopy for single-cell segmentation and counting. Alternative imaging modalities that capture the entire larval brain volume may be required to precisely inventory cell numbers in control vs As-exposed tissues. Alternatively, As-exposure may alter other cellular lineages not examined in this study.

Prior work confirms that As arrests the cell cycle at the G1/S and G2/M transition points due to decreased E2F1 transcriptional activity involving the retinoblastoma tumor suppressor (Sheldon 2017). Our findings are consistent with these results. While our data suggest As-exposed NSCs proceed through mitosis more slowly; in some contexts, aneuploid NSCs can trigger cell cycle exit and premature differentiation (Gogendeau *et al*. 2015). Further, some animals with aneuploid NSCs fail to complete larval development (Gatti and Goldberg 1991). The *Drosophila* brain is an ideal toolkit to investigate the neurodevelopmental consequences of early As-exposure.

As is commonly used in the laboratory to trigger the cell stress response, leading to the induction of stress granules, translationally repressed ribonucleoprotein complexes comprising RNAs and proteins, some of which are themselves associated with the cell cycle (Jain *et al*. 2016; van Leeuwen *et al*. 2022). Therefore, sequestration of cell cycle factors within stress granules is another mechanism by which arsenic contributes to cell cycle blockades. Within NSCs, delays in cell cycle progression are expected to alter neuronal specification, raising the likelihood that larval exposure to As may impact neurogenesis of escaper adults. While our low-dose experiments with As did not reduce NSC, glia, or neuron number, an area for future study would be to examine dose-dependent consequences to neuronal lineages.

In adult flies, chronic arsenic exposure leads to neurologic impairment, reducing locomotor activity as assessed by an NGA. Our data show that *Tau^R406W^* females perform more poorly in the climbing assay than their male siblings, but As-exposure did not worsen this effect. One model that could explain these findings is that As and the *Tau^R406W^* mutation impair climbing activity through the same pathway.

Notably, As impaired circadian rhythms primarily in *Tau^R406W^* females. This sex-specific response of *Tau* mutants is unintuitive given our LD50 calculations show females are more resilient to As-induced lethality, as recently reported for *Ore-R* (Holsopple *et al*. 2023). Unexpectedly, we detected sex-specific responses in *yw*, but not *Ore-R*. Why we failed to detect the sex-specific response in *Ore-R* is not known, but possible reasons for this discrepancy may include differences in sample sizes, As-concentrations tested in the refined LD50 experiment, or genetic drift of our *Ore-R* stock, maintained in our laboratory for many years.

Why female flies possess higher tolerance to the acute effects of arsenic toxicity is unknown, but their larger body size may be a contributing factor. Prior work also indicates that specific regions on chromosome 3 and the X chromosome mediate resistance to some external toxins, such as octanoic acid (Jones 1998). Whether similar genetic regions provide the mechanistic basis for why specific genotypes and sexes show differential responses to As warrants further study.

Nevertheless, our data support the idea that *Tau^R406W^* mutants are sensitized to As-exposure, at least with respect to sleep responses. While a similar toxicogenetic interaction was not observed with the NGA, whether other neurological responses are affected in *Tau* mutants exposed to As also warrants further study. It is feasible that specific neurologic pathways are more prone to As-toxicity than others.

Within the research setting, As is commonly used to stimulate the assembly of aggregation-prone proteins, including those associated with FTD, like Tau (Jacobson *et al*. 2012; Brunello *et al*. 2016). Thus, our work is consistent with the idea that As-exposure is deleterious to genetically sensitized neurodegenerative models. However, additional study of As-exposure risks and health outcomes in other neurodegeneration models and human ADRD patients should be explored.

## Supplementary Material

jkaf049_Supplementary_Data

## Data Availability

All data are available in the published article and its online Supplementary material, which includes 4 videos of NSC divisions and source data of 22 NGA movies: doi.org/10.6084/m9.figshare.26485753.
