# Supplementary material for: Arsenic impairs Drosophila neural stem cell mitotic progression and sleep behavior in a tauopathy model
Source: G3 (Bethesda). 2025 Apr 7;15(5):jkaf049. doi: 10.1093/g3journal/jkaf049 (PMC12060243; doi:10.1093/g3journal/jkaf049)
Supplement: jkaf049_Supplementary_Data [file jkaf049_supplementary_data.docx]

**Supplemental NSC Video Legends**

(Available at <https://doi.org/10.6084/m9.figshare.26485753>)

**Video 1. A control NSC expressing *H2AV-RFP* undergoing successive division cycles.** Images were acquired at 1-minute intervals for 69 minutes. The video is displayed at a playback speed of 7 frames per second (FPS). This video corresponds to stills from Figure 5A.

**Video 2. An As-exposed NSC expressing *H2AV-RFP* undergoing successive division cycles.** Images were acquired at 1-minute intervals for 109 minutes. The video is displayed at 7 FPS. This video corresponds to stills from Figure 5A.

**Video 3. A dividing control NSC expressing *H2AV-RFP*.** Images were acquired at 1-minute intervals for 4 minutes. The video is displayed at 7 FPS. This video corresponds to stills from Figure 5B.

**Video 4. A dividing As-exposed NSC expressing *H2AV-RFP.*** Images were acquired at 1-minute intervals for 6 minutes. The video is displayed at 7 FPS. This video corresponds to stills from Figure 5B.
